# Centering Youth Voice in the Adaptation of an mHealth Intervention for Young Adults With HIV in South Texas, United States: Human-Centered Design Approach

**DOI:** 10.2196/60531

**Published:** 2025-04-08

**Authors:** Nhat Minh Ho, Catherine Johnson, Autumn Chidester, Ruby Viera Corral, Jacundo Ramos, Miguel Garcia, Rishi Gonuguntla, Cyrena Cote, Divya Chandramohan, Hueylie Lin, Anna Taranova, Ank E Nijhawan, Susan Kools, Karen Ingersoll, Rebecca Dillingham, Barbara S Taylor

**Affiliations:** 1 Joe R. and Teresa Lozano Long School of Medicine, The University of Texas Health Science Center at San Antonio, San Antonio, TX, United States; 2 Department of Public Health, Innovation and Equity, University Health, San Antonio, TX, United States; 3 Department of Women’s Health, Dell Medical School, The University of Texas at Austin, Austin, TX, United States; 4 Division of Infectious Diseases, Department of Medicine, The University of Texas Health Science Center at San Antonio, 7703 Floyd Curl Dr., MSC 7881, San Antonio, TX, 78229, United States, 1 2105674661, 1 2105674670; 5 Department of Psychiatry, Washington University School of Medicine in St. Louis, St Louis, MO, United States; 6 Division of Infectious Diseases, Departament of Medicine, The University of Texas Southwestern Medical Center, Dallas, TX, United States; 7 School of Nursing, University of Virginia, Charlottesville, VA, United States; 8 School of Medicine, University of Virginia, Charlottesville, VA, United States

**Keywords:** HIV, implementation science, youth, mHealth, adherence, young, mobile health, mobile health intervention, AIDS, US, adult, self-efficacy, willingness, health outcomes, mHealth intervention, interview, human-centered design, acceptability, usability, mobile phone

## Abstract

**Background:**

Young adults living with HIV are less likely to engage in care and achieve viral suppression, compared to other age groups. Young adults living with HIV also have a high degree of self-efficacy and willingness to adopt novel care modalities, including mobile health (mHealth) interventions. Interventions to increase care engagement could aid young adults living with HIV in overcoming structural and social barriers and leveraging youth assets to improve their health outcomes.

**Objective:**

The objective of the paper was to use an assets-based framework, positive youth development, and human-centered design principles to adapt an existing mHealth intervention, PositiveLinks (PL), to support care engagement for 18‐ to 29-year-olds with HIV.

**Methods:**

We conducted a formative evaluation including semistructured interviews with 14 young adults with HIV and focus groups with 26 stakeholders (providers, nurses, case managers, and clinic staff). Interviews covered barriers to care, provider communication, and concerns or suggestions about mHealth interventions. The research team used thematic analysis to review interview transcripts. In the second phase, human-centered design processes informed adaptation of the existing PL platform using data from real-time use suggestions of 3 young adults with HIV. Throughout the formative evaluation and adaptation, a Youth Advisory Board (YAB) provided input.

**Results:**

Young adults with HIV and stakeholders identified common elements of an mHealth intervention that would support care engagement including: the convenience of addressing needs through the app, online support groups to support interconnection, short videos or live chats with other young adults with HIV or providers, appointment and medication reminders, and medical information from a trustworthy source. Stakeholders also mentioned the need for youth empowerment. Concerns included worries about confidentiality, unintentional disclosures of status, urgent content in an unmoderated forum, and the impersonality of online platforms. Design suggestions from young adults with HIV included suggestions on appearance, new formatting for usability of the online support group, and prioritization of local content. Based on the feedback received, iterative changes were made to transform PL into Positive Links for Youth (PL4Y). Final votes on adaptations were made by the YAB. The overall appearance of the platform was changed, including logo, color, and font. The online support group was divided into 3 channels which support hashtags and content searches. The “Resources” and “Frequently Asked Questions” sections were condensed and revised to prioritize South Texas–specific content.

**Conclusions:**

Our assets-based framework supported young adults with HIV and stakeholder input in the transformation of an mHealth intervention to meet the needs of 18- to 29-year-olds in South Texas. The human-centered design approach allowed young adults with HIV to suggest specific changes to the intervention’s design to support usability and acceptability. This adapted version, PL4Y, is now ready for pilot testing in the final phase of this implementation science project.

## Introduction

### Background

Young adults living with HIV face difficulty with care engagement [1]. While existing studies of young adults with HIV include varying age ranges, spanning 13 to 29 years, most agree that young adults with HIV across the age spectrum face unique challenges in care engagement. These include limited opportunities for care, higher rates of missed appointments, and medication nonadherence, all of which contribute to difficulties in achieving viral suppression [2-4]. Interventions to increase care engagement could help young adults with HIV overcome structural and social barriers to improve health outcomes. While young adults with HIV often seek mobile health technologies to support care engagement, few were developed or adapted with their input or needs in mind.

Most young adults with HIV have a high degree of self-efficacy and willingness to adopt novel care modalities, particularly technological innovations [5]. In 2018, a national survey revealed 45% of adolescents report being on the web almost constantly, and 95% of teens own a smartphone [6]. Mobile health (mHealth) interventions are well suited to these digital natives. Data show that mobile phone apps are an acceptable and often preferred way for adolescents to receive sexual health information and successfully engage those susceptible to HIV [7-10]. Considering the developmentally-appropriate desire for self-determination found among young adults with HIV, they may appreciate mHealth interventions offering an expanded range of services, particularly functions that support self-management of HIV and digital connections [11,12]. However, the majority of current mHealth interventions offer a limited number of functions, such as medication or appointment reminders [7,8,10,13].

### mHealth Interventions and Care Engagement for Young Adults With HIV

A few mHealth interventions focusing on young adults with HIV have shown promising results [9,10,13]. A mobile intervention that included peer navigation and a webcomic series increased entry to care, decreased rates of loss to follow up, and supported sustained viral load suppression for young adults with HIV [9]. A review of mHealth intervention modalities for 18- to 34-year-old people living with HIV found advantages such as improved accessibility to information, enhanced self-efficacy, and reduced negative feelings about HIV diagnoses [13]. Other forms of mHealth interventions previously used in promoting the health of young adults with HIV include remote coaching, short message services, phone calls, and social media outreach [14,15]. mHealth intervention implementation faces the challenge of needing continuous updates based on input from stakeholders and app users [10].

Few studies in the existing literature describe how mHealth interventions can be tailored to the needs of young adults with HIV or adaptation processes that ensure they play an active role in intervention design. The Positive Youth Development (PYD) framework suggests that youth involvement and empowerment can lead to better health and reduced risk-taking behaviors [1,16,17]. Aligned with PYD, peer empowerment, knowledge seeking, and taking responsibility for health outcomes improve engagement in health-promoting processes and resilience among gay and bisexual youth with HIV [18]. These data suggest that the PYD framework may be used to incorporate youth input into intervention design and, ideally, lead to more impactful interventions.

Young adults with HIV in South Texas experience significant barriers to care engagement for HIV, making the region an important location to study innovative interventions [19]. In 2016, the US Centers for Disease Control and Prevention (CDC) began using phylogenetic analysis to identify geographic areas where HIV transmission and new diagnoses were more likely. The first iteration of this analysis identified several clusters of new diagnoses in Texas, the largest of which was in South Texas, where 78% of the individuals newly diagnosed were under 30 years old. The majority were also uninsured, without access to HIV prevention services, and Hispanic or Latino [20,21]. Other literature suggests that young men who have sex with men of color experience structural racism, which may alienate them from health care services [4,22]. Despite these challenges, South Texas also has a strong history of collaborative work between community clinics, the county health system, and the local health authority to end the HIV epidemic [23,24]. These factors make the region an ideal site to create and implement a new mHealth intervention targeted specifically toward young adults with HIV.

### Investigation Goals and Significance

The aim of this investigation is to collaborate with young adults with HIV and create a space for their voices and input to adapt an existing mHealth intervention, PositiveLinks (PL), to improve care engagement in 18- to 29-year-old people living with HIV in South Texas [25]. This age range was selected as they experience higher rates of new diagnoses and a lower prevalence of sustained viral suppression [4]. PL is a clinic-based multimodal HIV care intervention that increases care engagement and viral suppression [25]. The platform includes daily medication adherence tracking, mood and stress self-assessment, appointment reminders, secure messaging with clinic providers, and an anonymous online peer support network [25]. We used human-centered design thinking processes in this adaptation to ensure young adults with HIV input, as interventions are more effective when impacted communities are involved in their design and implementation [5].

We conducted a formative evaluation using data from semistructured interviews with young adults with HIV and stakeholders, as well as focus groups with stakeholders to understand acceptable and desired intervention characteristics [19]. Subsequently, we used design thinking processes to aid in our adaptation of PL [16]. This paper describes findings from each phase to enhance understanding of the components of mHealth interventions that are accepted and sought after by young adults with HIV.

## Methods

### Overview

**Figure 1.**
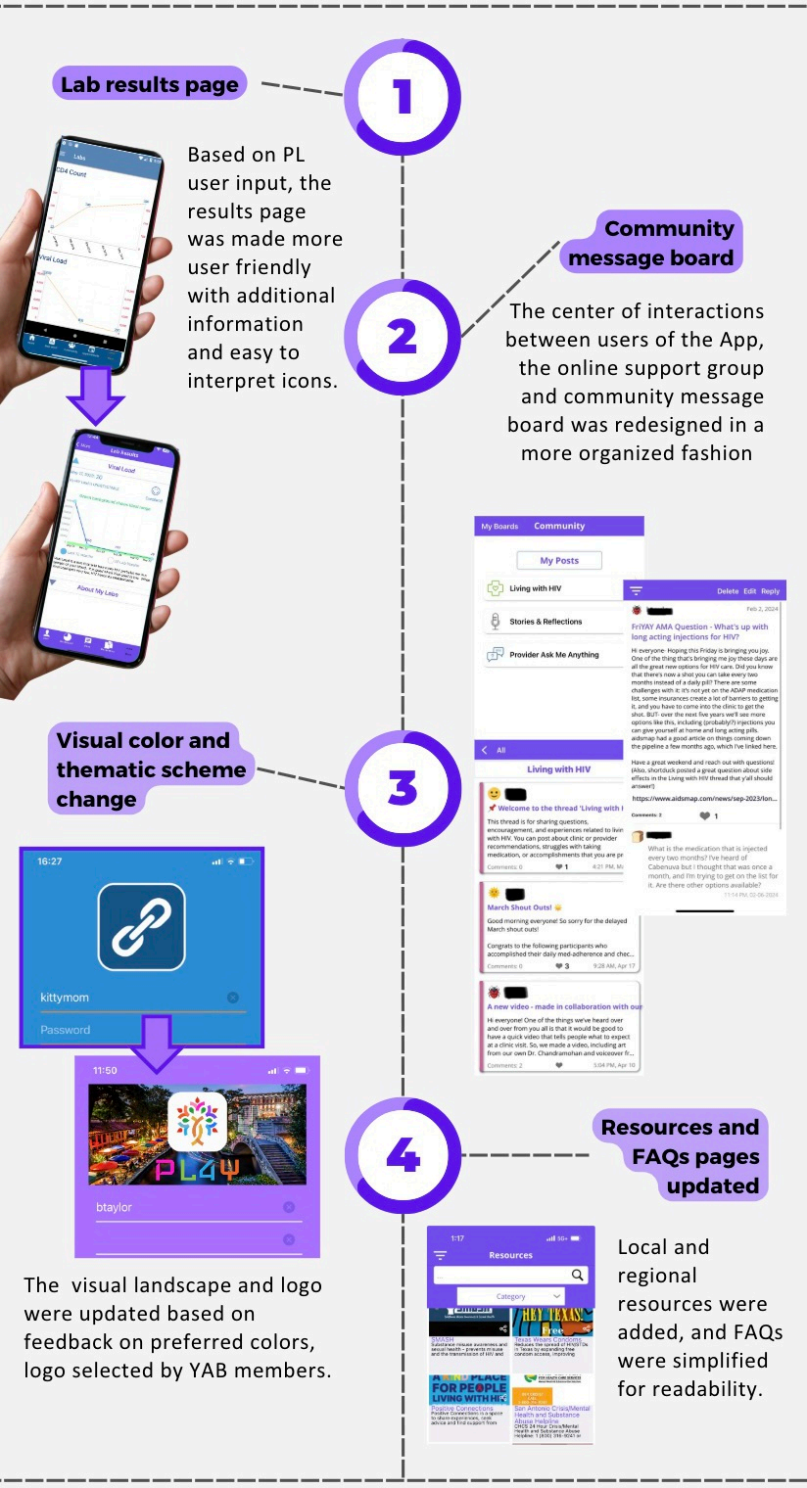
Triangulation of data sources for adaptation of the mobile health (mHealth) intervention to the needs of young adults with HIV in South Texas. PL: PositiveLinks; YAB: Youth Advisory Board; FAQ: frequently asked questions.

Adaptation of the existing mHealth intervention, PL, into its novel iteration, Positive Links for Youth (PL4Y), was informed by three data sources: (1) components of the formative evaluation that addressed acceptable mHealth interventions, (2) a human-centered design thinking process, and (3) A Youth Advisory Board (YAB) of young adults with HIV that provided input throughout the formative evaluation and adaptation phases (Figure 1). There was no overlap in membership between the young adults with HIV participating in interviews and the YAB during the formative evaluation. A total of 3 young adults with HIV interviewed during the formative evaluation joined the YAB for subsequent phases of the project, the human-centered design thinking process, and the pilot intervention with the adapted PL4Y app, currently underway. The project used the Consolidated Framework for Implementation Research (CFIR), gathering knowledge of individuals involved and the inner and outer settings surrounding the intervention from a formative evaluation, and then iteratively adapting the intervention to fit the context [26]. A PYD framework also informed study activities, which incorporated input of young adults with HIV in the form of interviews, active feedback during the human-centered design thinking process, and the YAB [11,12]. All 3 components were overseen by the design working group, which included the principal investigator (PI), study coordinator, research assistants, and the scientists, clinic staff, and software designers on the original PL team.

### Recruitment

Members of the YAB were recruited through meetings with community-based organizations, flyer distribution in HIV service organizations and clinics, and collaboration with advocates for young adults with HIV. For the formative evaluation phase and Think-Aloud interviews, young adults with HIV were recruited from 6 HIV treatment centers in South Texas using flyers and direct outreach to clinic staff and providers. A total of 14 participants were enrolled in the formative evaluation phase. Of the 3 participants in the Think-Aloud session, 2 had previously participated in the formative evaluation phase. Inclusion criteria required members to be 18- to 29-year-old and living with HIV in San Antonio, Texas. Stakeholders from 6 regional HIV treatment centers were also recruited for the formative evaluation phase.

### Youth Advisory Board Role and Input

PL4Y began with the creation of a YAB consisting of 18‐ to 29-year-old people living with HIV in South Texas [1,19]. As described previously by Chidester et al [1], the YAB’s structure and format evolved in response to the COVID-19 pandemic and feedback from YAB members. YAB engagements occurred quarterly, with input collected through Qualtrics surveys to preserve YAB member anonymity.

**Figure 2.**
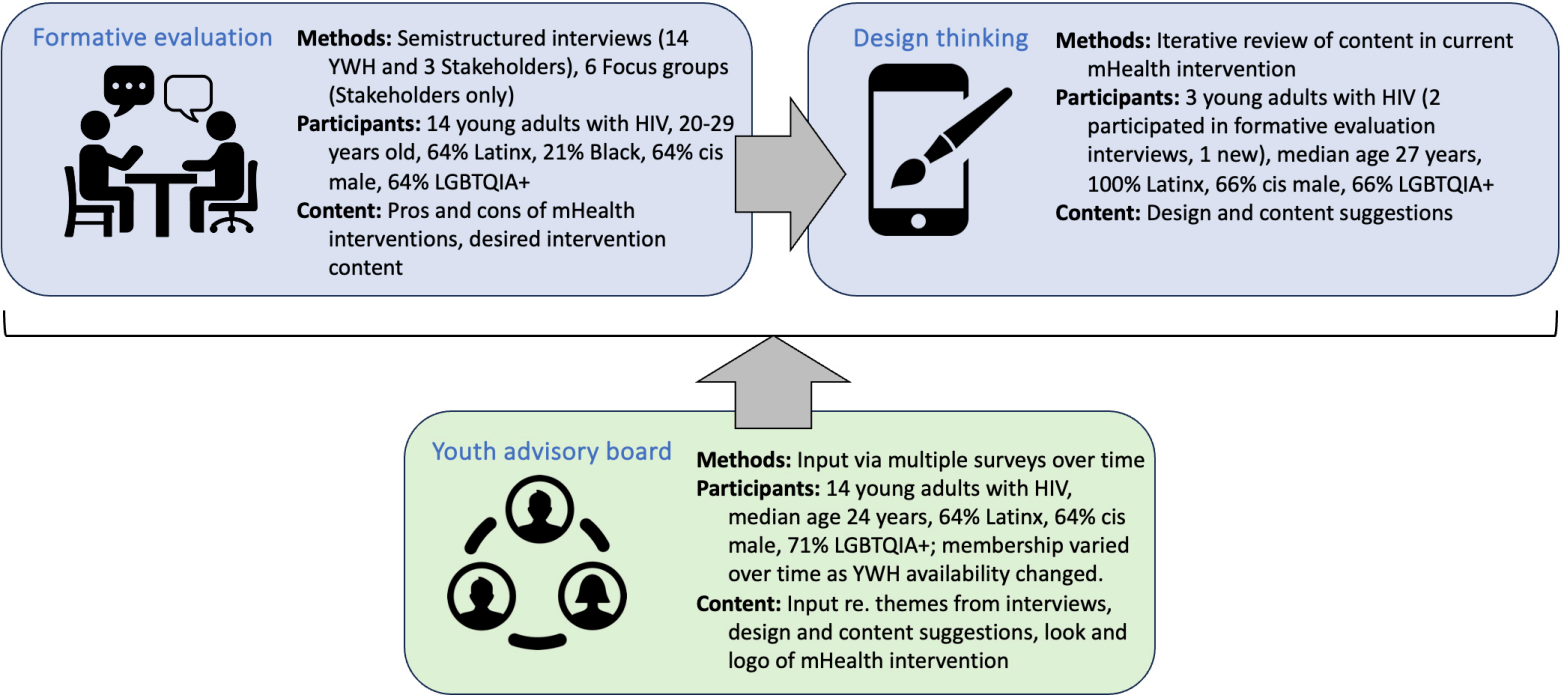
Overview of 4 human-centered design thinking driven adaptations to the existing intervention to respond to expressed needs of young adults with HIV during Think-Aloud sessions and the Youth Advisory Board (YAB). mHealth: mobile health; LGBTQIA+: lesbian, gay, bisexual, transgender, queer, intersex, asexual, and other minoritized gender and sexual identities.

Topics covered in the surveys included desired elements of an mHealth intervention, topics for the community message board, preferred mobile app security options (password, fingerprint, face ID, etc), and feedback on the design and look of the app. In the final stage of design iteration, responses regarding the graphics and the app interface from previous YAB surveys and the Think-Aloud sessions were reviewed by the design working group. The software developers incorporated this feedback into the design of a new look and logo for PL4Y. A Qualtrics survey was distributed to the YAB, asking respondents to rate and rank potential logos and designs (N=11). The top 3 logos and fonts (determined by average ranking and average rating) were implemented into a second Qualtrics survey (N=9). The highest average ranked combination of logo and font was selected as the official logo of PL4Y (Figure 2).

### Formative Evaluation of Acceptable mHealth Intervention Components

The formative evaluation of care engagement for young adults with HIV in South Texas was conducted from October 2020 to December 2021. Eligibility criteria included being 18‐29 years of age, residing in South Texas, fluency in either English or Spanish, and reporting current or past challenges with care engagement. Stakeholders included 26 office, nursing, and provider staff members from the regional HIV treatment centers, who provided input either through 6 focus groups or 3 individual interviews.

Interview and focus group guides were developed based on the theory of planned behavior (TPB) theoretical model, the PYD framework, and additional themes provided by the YAB. young adults with HIV were asked about first-hand experiences that affect their care engagement as young people residing in South Texas. Stakeholders were asked to describe their experiences and beliefs regarding serving young adults with HIV and to provide system-level insights on strategies for improving care engagement. To reduce bias and support generative reflection on desired components, neither young adults with HIV or stakeholders were provided with access to the original version of the PL app. A transcription service was used to transcribe individual interviews and focus groups. Transcripts were reviewed and edited by the interviewer (CJ) for accuracy. Transcripts were uploaded into NVivo 1.6.2 (Lumivero), which allows for tracking of responses by stakeholder or young adults with HIV characteristics and by clinic site.

The qualitative analysis team (QAT) created a master codebook of themes derived from TPB and PYD theoretical frameworks for thematic analysis [1,16,17]. The QAT included 4 research assistants with training in qualitative analysis (CJ, AC, HL, and NMH), 5 faculty-level researchers with expertise in HIV care engagement (AT, AN, KI, RD, and BT), and 1 youth development expert (SK). A member of the primary coding team independently coded transcripts inductively (CJ, AC, and HL), with review by the QAT lead (BT). Secondary coders (CJ, AC, HL, and NMH) expanded on coding as needed. At this stage, the QAT also examined themes by participant type and noted differences between young adults with HIV and stakeholder perspectives. The QAT resolved differences in coding by consensus and confirmed revisions to codebooks. The full QAT participated in the final round of coding, where all themes were reviewed, representative quotes discussed, and themes were renamed, merged, or discarded. Overall findings from the formative evaluation regarding care engagement for young adults with HIV in this context were the topic of a separate analysis [19]. This analysis reviewed themes on advantages and disadvantages of mHealth interventions in general and themes regarding specific mHealth intervention components desired by young adults with HIV or stakeholders.

### Human-Centered Design Adaptation of Positive Links by Young Adults with HIV

A total of 3 Think-Aloud interviews were conducted with young adults with HIV using human-centered design principles to ensure end-user input informed an iterative adaptation process [17,18]. A total of 2 Think-Aloud sessions were conducted in person at a local clinic, and 1 was conducted online through Zoom. In addition, 3 researchers (CJ, AC, and NMH) were present for interviews and a modified system usability scale guided conversation [27]. The Think-Aloud participants reviewed each aspect and function of a previous version of the app by walking through it on their phones in real time, providing suggestions for possible design changes and usability improvement. Adaptation recommendations were categorized by ease of implementation and frequency. The most common and feasible suggestions were conveyed to the University of Virginia app development team for final design and development.

### Ethical Considerations

All young adults with HIV and stakeholders participating in the formative evaluation and human-centered design components reviewed an institutional review board (IRB)–approved information sheet about the study and provided verbal consent to participate. Participants in the focus groups and Think-Aloud sessions received a US $25 electronic gift card. Stakeholders did not receive monetary incentives for participation. YAB members also received a US $25 electronic gift card upon completion of each survey. This protocol was approved by the UT Health San Antonio and University Health IRBs (HSC20220752HU)

## Results

### Acceptability and Concerns Regarding mHealth Interventions From the Formative Evaluation

Qualitative data from young adults with HIV and stakeholders revealed common themes about benefits and challenges of an mHealth care engagement intervention. Overall, young adults with HIV and stakeholders felt ease of access to resources, labs, and clinic personnel would be beneficial, and that the anonymous online support group could help young adults with HIV feel less isolated. However, concerns were raised about confidentiality on an mHealth platform and potential pitfalls of the online support group, including the need for moderation and issues with web-based connections (Textbox 1).

Textbox 1. **Summary of perceived benefits, challenges, and critiques of an mHealth care engagement intervention expressed by 18- to 29-year-olds living with HIV and stakeholders.**

Benefits:

- Increased accessibility of care team.
- Increased access to accurate information about health issues and available resources.
- App-based medication and appointment reminders.
- Ability to track treatment progress through improvements in lab parameters.
- Improved access to a safe community through a online support group.
- Anonymity provided by online support group platform.

Challenges:

- Potential confidentiality concerns.
- Lack of streamlined features.
- Difficulty connecting to other members in the online setting.
- Need for moderation of online support group with potential for cyberbullying.
- Live chat could trigger negative or apprehensive feelings.

### Perspectives on Benefits of the mHealth Intervention

#### Accessibility to Care and Knowledge

Youth and stakeholders believed the app would be highly effective in connecting patients with providers and case managers. Young adults with HIV noted that having an app on their smartphone could increase accessibility to their care team, making them more likely to participate in their care. They also emphasized the app could help them understand what programs they were eligible for and how to apply for support without visiting a clinical setting.

You’ll be more likely to actually participate in that if it’s, you know, easy. It’s, like, virtual; so that’s a big thing.

[Youth Voice [YV] 8]

If you have a phone or some type of smart device, you would be able to access it wherever you are.

[YV5]

Stakeholders further noted the online nature of the app provides a unique benefit, particularly in the COVID-19 era, by enabling patients to connect with their support network without meeting in person. They believed this benefit extended beyond the pandemic, as it also improves access for patients in rural settings.

We don’t know how many people are at home alone.

[Stakeholder Voice [SV] 9]

Furthermore, young adults with HIV and stakeholders felt the app could centralize trustworthy information and serve as an assessable resource.

You may not want to ask the doctor, but just, like, you want a quick question answered.

[YV6]

#### Advantages of an Online Support Group

Youth recognized the benefits of an online support group, describing it as a safe community where people with similar life experiences could connect. They noted that such a group could reduce stigma by fostering a sense of camaraderie.

I think with a support group, it’s, like, knowing that you’re not alone and that, like, it’s not a end-of-the-world thing; and you’re not dying. You know, you have other people who are living with it and surviving; and it kind of decreases that stigma because you know that there are millions, you know, that are dealing with the same issue.

[YV6]

Both young adults with HIV and shareholders appreciated the anonymity provided by an app-based support group. Young adults with HIV emphasized the app’s online platform offered privacy, as they would not need to visit a physical location to engage with a support group.

It’s good because it’s like I don’t have to go and park there to go see somebody.

[YV13]

Stakeholders believed newly diagnosed patients would be able to ask about symptoms without feeling guilt. In addition, they highlighted that the anonymity of the app may enable more people to feel comfortable contributing to the community without fear of being judged.

Like if they want us to stay a little bit more anonymous but have some place where they can talk to some other people that are going through something similar, or to be like, hey, I’m having these weird symptoms, anybody else experienced that or whatever.

[SV25]

#### Reminders

Young adults with HIV noted app-based medication reminders would have been helpful when they were first diagnosed and had not already established a medication routine.

I think it would be good for other people that are barely starting, so they can remember to drink their meds.

[YV3]

Young adults with HIV and stakeholders reported they would welcome medication refill reminders and appointment reminders.

Oh, that would definitely be helpful—medication reminders for sure because I will go to my very last, like, tablet; and I will forget, and then it’s a hassle.

[YV6]

Stakeholders identified that reminders could provide encouragement by tracking treatment progress and enabling users to share their milestones within a community. This, in turn, would empower young adults with HIV to continue engaging in care.

Hey, your viral load was this high and look how it is now. I think it would be helpful, you know, when they become undetectable or they can, you know, share with partners.

[SV18]

Young adults with HIV reported they wished to have “testimonials/videos of people living with HIV,” and “facilitated conversations” on the app.

### Disadvantages of an mHealth Intervention

#### Confidentiality and Anonymity

Although the app’s presence on a smartphone increases accessibility, young adults with HIV and stakeholders expressed concerns about potential for unintentional disclosure if someone else sees the app on their device. Both groups identified notifications as a particular risk for exposure.

Somebody else gets access to your account, and I mean, that’s biggest my fear… Maybe getting notifications when I didn’t want to get a notification and it kind of exposing me.

[YV7]

#### Design Critiques

Some young adults with HIV and stakeholders mentioned having too many settings in the app may limit its usability. They wanted a streamlined design with access to educational content. In addition, youth expressed concerns about potential redundancy in notification, where the same message might appear in multiple locations on the smartphone.

It’s just redundant when I’m using the same device over and over again.

[YV6]

Young adults living with HIV and stakeholders voiced concerns that online interactions could feel impersonal. A participant reported negative experiences with previous web-based communities of people living with HIV.

But I just never really, I never really identified—or like connected with any of them. They were … people from (other countries). But no Hispanic people.

[YV11]

Other young adults living with HIV reported that online resources did not feel genuine and preferred in-person interactions.

Going to an actual in-person group, having the actual in-person interaction with people for me would have more of an impact than just kind of talking to someone on my phone.

[YV5]

Despite the potential impersonal experience, stakeholders pointed out that people are more accustomed to virtual settings than in the past.

But a lot of things just nowadays is really virtual, just like what we’re doing here today. You know, a lot of it is just really impersonal.

[SV3]

#### Concerns for Unmoderated Interactions

Young adults living with HIV were concerned that without moderation, there was the possibility of smaller communities developing within mHealth interventions and online communities, making the platform less approachable for new users. In addition, concerns were voiced about the lack of moderation allowing users to write irrelevant or offensive posts within the forum. A participant described their experience reading comments on a thread for a online discussion group:

Every time you scroll down and you think you’re done, there’s like 20 more in the bottom and they’re just saying random stuff.

[YV9]

Young adults living with HIV and stakeholders had concerns regarding the potential for “trolls” or “cyberbullying.” They were particularly concerned about those who would be able to download and join the app community following implementation.

Maybe if a certain type of group of people get ahold of it… They have easy access to basically come on and troll everybody.

[YV14]

Stakeholders additionally expressed concerns about delayed responses to urgent messages written in the live chats, given staff are not constantly monitoring the service. However, they believe this risk could be mitigated by educating patients that urgent messages should not be conveyed through the app and that messages will be reviewed within a designated time.

Patients may just send the chat like saying… I have fever and shortness of breath. And then Friday at 5:30 and nobody’s there in the chat feature. So, I think that that’s a little bit dangerous.

[SV18]

Young adults with HIV voiced apprehension about how video support groups or live chats would affect them. A user expressed they might experience a, “feeling of insignificance, just because maybe somebody is going through it worse than you are” (YV2). In addition, young adults living with HIV stated concerns about how listening to other people’s testimonies may, “make it more real for them” (YV5).

and it is unpleasant if people minimize their experience on the app. They also speculated that users interested in building connections may migrate to other platforms, such as social media, for deeper conversations.

But I don’t think that they’re going to really utilize just this App to message back and forth. Because they’ll be like, “Hey, you’re cool.” “Oh, you’re cool, too. Here’s my Instagram.” And then they’re going to use that.

[YV13]

### Design Suggestions From Young Adults With HIV Derived From Think-Aloud Sessions

Young adults with HIV wanted to know who would have access to the information they put into the app, such as medication adherence, mood, or stress. App users can report daily medication adherence, stress levels, and mood “check ins” to log their emotions. Think-Aloud participants emphasized the importance of ensuring that no one views their data without consent.

Participants provided specific suggestions to improve the app’s aesthetic appeal, requesting a more “modern look.” They had strong opinions against the original color scheme of the app and the old logo, which featured two chain links. The original blue color scheme was deemed “too medical” by participants, who suggested either a “plain” or “vibrant” color scheme. Participants were quick to point out incorrect scaling on thumbnails and other visually unappealing color schemes that reduced the visual charm of the original PL app.

During Think-Aloud sessions, participants reported certain design aspects were nonintuitive, hindering the ease of use. Participants suggested reorganizing the community message board from a single thread to allow participants to self-organize threads, optimizing the search bar for resources and FAQs, having convenient “learn more” hyperlinks within lab results, and implementing “read receipts” to confirm whether messages and documents sent were seen by all users, including health care providers.

Participants expressed particular interest in finding local resources relevant to South Texas. They were eager to learn about the resources offered within the app but suggested making them more community specific. Participants also found the FAQ section useful for patients newly diagnosed with HIV and suggested including a glossary of acronyms to help understand frequently used terminology in HIV care. Participants also emphasized the need for customizability and improved integration within their devices. Specifically, they requested ways to customize the app’s home screen icon and to modify notification settings to allow for privacy.

Several suggestions made by Think-Aloud participants were determined to be beyond the scope of the current adaptation, but important to consider in the future. These potential adaptations include saving posts, blocking specific users, and prioritizing conversations within community message boards. Young adults living with HIV also requested the ability to add photos to posts, replies, and messages. Participants additionally suggested new features for access to care, such as online bus passes.

### Adaptation Process

The design working group incorporated findings from the formative evaluation and Think-Aloud sessions into the app’s redesign. The laboratory section was updated to be more understandable for young adults with HIV. The community message board was divided into 3 distinct channels, allowing members to “like” other posts, and implementing a search by word or hashtag function. The color scheme, font, and logo were updated based on feedback by the YAB, as described above (Figure 2). The “Resources and FAQ” section was condensed and revised to prioritize material relevant to South Texas. This adaptation process is ongoing as we continue to add requested content, such as videos explaining the steps to an HIV clinic visit.

## Discussion

### Principal Findings

We incorporated the voices of young adults with HIV throughout the process of adapting an mHealth care engagement intervention using an implementation science–informed approach. This process led to significant changes in the intervention. Young adults with HIV identified several advantages of mHealth interventions, including ease of access, community building, and medications and appointment reminders. However, they also voiced concerns about confidentiality, the impersonal nature of online interactions, and lack of content moderation. Based upon feedback from the formative evaluation, Think-Aloud sessions, and the YAB, the intervention was adapted to meet expressed needs. Modifications included changing the graphic design and logo, adding South Texas specific resources, updating visualization of laboratory data, and designing a more user-friendly and youth-appropriate community message board.

The medical literature suggests young adults with and those without HIV accept app-based mHealth interventions, which can be used to improve health behaviors for youth and emerging adults [9,10,13,28]. An app-based intervention was found to be successful in an adolescent population with asthma, as well as in an adolescent population in recovery following hematopoietic stem cell transplant [29,30]. There have been few studies investigating the usability of app-based interventions in patients with HIV. Aladin et al [9] enrolled 113 patients with HIV and found their app-based intervention to be associated with increased communication and engagement with care when combined with peer navigation and a comic series. However, the majority of mHealth interventions are text message–based between patient and clinic, and users lack the ability to form connections with each other [31]. Brooks et al [13] found that app-based interventions were most useful for young adults with HIV if they had medical information and reminders, leading to increased self-efficacy, supported feelings of connection to a community, and reduced stigma around HIV. These results indicate users of app-based interventions may benefit from “discussion board” components that allow users to communicate with one another and develop a community. Schnall et al [32] studied the functions of 15 publicly available app-based interventions targeting people living with HIV and found 4 enabled communication between peers and providers, 6 had medication reminders, 7 had medication logs, and 6 included a search function [32]. This analysis aligns with the existing literature as both users and stakeholders identified all of the above as critical functionalities of an app-based intervention, and these components were included in the final redesign.

Our findings add to this existing knowledge base by highlighting the importance young adults with HIV place on easy, asynchronous access to services. Young adults with HIV welcome the ability to message providers or other members of the care team, quick links to web-based resources for HIV care and social services, and automatic appointment and medication reminders. We discovered many young adults with HIV wished for connections to other young adults with HIV, something not currently featured in mHealth interventions for this demographic. They also appreciated the online, anonymous format of the community message board. Finally, young adults with HIV repeatedly emphasized that the graphic design and usability of the intervention should be influenced by what was most acceptable and appealing to them. These data influenced our own design adaptation of PL into PL4Y but are also relevant to framing other mHealth interventions for young adults with HIV or other youth communities.

Few studies investigate patient thoughts regarding current mHealth interventions or discuss the concerns of young adults with HIV with this intervention modality. A review of 7 mHealth interventions by Conserve et al [33] found cellular network restrictions were a significant barrier to the use of mobile technologies for young adults with HIV. Our findings echo this, as both young adults with HIV and the stakeholders anticipated access to a smartphone could be a barrier to use of our intervention.

We found additional young adults with HIV concerns with mHealth intervention that, to our knowledge, are not reflected in the existing literature. Many young adults with HIV expressed concerns about confidentiality and security of the data on their phone. Others worried that communicating by electronic messaging, rather than in person or by phone call, would feel impersonal. Young adults living with HIV were also concerned with exposure to harmful or disempowering narratives on the community message board. Though our intervention already has robust security and content monitoring protocols, these concerns will need to be addressed during implementation.

### Limitations

Despite the meaningful findings of this investigation, there are limitations. Our adaptation process incorporated viewpoints of a limited number of young adults with HIV participants and YAB members. While their perspectives were valuable, we cannot assume they represent the perspectives of all young adults with HIV. Furthermore, our participants reflect the South Texas population, with the majority identifying as Latinx, which may limit the generalizability of our findings. Despite these limitations, this study fills a crucial gap in the literature by identifying crucial barriers and facilitators to the adoption of an app-based mHealth intervention for young adults with HIV. It also increases our understanding of the understudied South Texas community’s potential response to other mHealth interventions [34].

### Conclusions

mHealth interventions targeting young adults with HIV can improve care engagement and health outcomes. However, few studies have identified the specific characteristics that promote or hinder patient adoption of app-based interventions [9,10,13,28]. Our adaptation process successfully incorporated input from a majority Hispanic or Latinx group of young adults with HIV in the design of a mHealth intervention. Our process demonstrated that young adults with HIV should play integral roles in the design of interventions for this population. We identified novel concerns not previously reflected in the literature including both barriers and facilitators to app-based mHealth interventions for young adults with HIV. We also describe a rigorous and iterative adaptation process that centers youth voice to ensure that the new intervention meets the needs of the community, successfully transforming PL into PL4Y. Currently, our team is testing the feasibility and acceptability of the adapted PL4Y mHealth intervention in a randomized control trial among young adults with HIV in South Texas. Our findings can also be used in the design of future app-based mHealth interventions to promote their adoption by young adults with HIV.

## Abbreviations

CDC: Centers for Disease Control and Prevention
CFIR: Consolidated Framework for Implementation Research
IRB: institutional review board
mHealth: mobile health
PI: principal investigator
PL: PositiveLinks
PL4Y: Positive Links for Youth
PYD: Positive Youth Development
QAT: qualitative analysis team
SV: Stakeholder Voice
TPB: theory of planned behavior
YAB: Youth Advisory Board
YV: Youth Voice
